# Enhancing the Antioxidant Potential of *Weissella confusa* PP29 Probiotic Media through Incorporation of *Hibiscus sabdariffa* L. Anthocyanin Extract

**DOI:** 10.3390/antiox13020165

**Published:** 2024-01-28

**Authors:** Natalia Simionescu, Anca-Roxana Petrovici

**Affiliations:** 1 Centre of Advanced Research in Bionanoconjugates and Biopolymers Department, “Petru Poni” Institute of Macromolecular Chemistry, 41A Grigore Ghica Voda Alley, 700487 Iasi, Romania; natalia.simionescu@icmpp.ro; 2 The Research Institute of the University of Bucharest (ICUB), 90 Sos. Panduri, 050663 Bucharest, Romania

**Keywords:** *Weissella confusa*, *H. sabdariffa* L. anthocyanins, antioxidant properties, ABTS and DPPH radical scavenging properties, hydroxyl radical and superoxide anion radical scavenging ability, FRAP assay, lipid peroxidation inhibitory activity

## Abstract

Lactic acid bacteria (LAB) produce important metabolites during fermentation processes, such as exopolysaccharides (EPS), which represent powerful natural antioxidants. On the other hand, *H. sabdariffa* L. anthocyanin extracts protect LAB and support their development. This study uncovers for the first time, the antioxidant profile of *Weissella confusa* PP29 probiotic media and focuses on elevating its impressive antioxidant attributes by synergistically integrating *H. sabdariffa* L. anthocyanin extract. The multifaceted potential of this innovative approach is explored and the results are remarkable, allowing us to understand the protective capacity of the fermented product on the intestinal mucosa. The total phenolic content was much lower at the end of the fermentation process compared to the initial amount, confirming their LAB processing. The DPPH radical scavenging and FRAP of the fermented products were higher compared to ascorbic acid and antioxidant extracts, while superoxide anion radical scavenging and lipid peroxidation inhibitory activity were comparable to that of ascorbic acid. The antioxidant properties of the fermented products were correlated with the initial inoculum and anthocyanin concentrations. All these properties were preserved for 6 months, demonstrating the promising efficacy of this enriched medium, underlining its potential as a complex functional food with enhanced health benefits.

## 1. Introduction

In the last three decades, countless research groups from all over the world have extensively studied all kinds of plant extracts. Plants that were known from traditional medicine to have healing properties were further studied for their biological activity and were tested in order to define their antimicrobial or anticancer activity. In this characterization stage, it was observed that the high antioxidant activity of extracts is generally correlated with a high antimicrobial and/or anticancer activity [1]. In addition, antioxidants represent molecules which are able to stabilize, deactivate, inhibit, and scavenge free radicals, thus protecting biological structures against oxidative damage [2,3]. Free radicals are generated when oxygen, as the last electron acceptor in the electron transport chain that produces ATP, becomes uncoupled [4]. Although all organisms possess the enzymatic equipment for antioxidant defense, the balance between ROS production and scavenging determines the body’s susceptibility to oxidative damage [5].

As major gut microbiota strains, lactic acid bacteria (LAB) produce important metabolites during fermentation processes, including bacteriocins and exopolysaccharides (EPS). Recent studies postulate the EPS potential in gut bacteria interaction, adhesion, colonization, and stress resistance, having a large contribution in health management [6]. In addition to these properties, EPS have many important biological properties such as immunomodulatory, anti-diabetic, cholesterol-lowering, antioxidant, and antitumor properties besides their roles in improving foods’ physicochemical characteristics [7]. For example, *Weissella* sp. biosynthesizes significant amounts of EPS and non-digestible oligosaccharides, which are compounds that can be used as prebiotic substrates or for other applications [8].

The available literature data report that LAB EPS are powerful natural antioxidants and are considered consumer-safe, promoting human health [8]. The EPS extracted from *W. cibaria* GA44 culture media exhibited very strong antioxidant properties, by scavenging superoxide anions and hydroxyl radicals, being a renewable natural source with antioxidant activity and promoting health benefits [8]. Some research groups demonstrated that EPS purified from *Lactobacillus plantarum* JLAU103 [6], *Lactobacillus plantarum* YML009, *Lactobacillus gasseri* FR4, or *Weissella cibaria* SJ14 [9] possess strong scavenging capacities against ABTS, DPPH, and hydroxyl radicals besides strong oxygen radical scavenging capacity and ferrous ions chelating activity. Adebayo-Tayo and Fashogbon showed that EPS biosynthesized by *Lactobacillus delbrueckii* subsp. *bulgaricus* had high antioxidant activity in a dose-dependent manner (DPPH radical scavenging, total antioxidant activity, H_2_O_2_ scavenging activity, and ferric ions’ reducing antioxidant power (FRAP)) [10]. Also, EPS from *Lactococcus lactis* subsp. *lactis* presented strong antioxidant properties in vitro and in vivo [11]. It was hypothesized recently that *Weissella cibaria* and *Weissella confusa* biosynthesize high amounts of EPS in order to protect cells against oxidative cytotoxic effects [12]. Overall, it can be concluded that EPS possess antioxidant properties with varying strengths, depending on their chemical structure and biosynthesizing microorganism.

*H. sabdariffa* L. (Malvaceae), also known as roselle, is an annual or perennial plant used around the globe as a drinking infusion, food, or medicine [13], being documented as early as 4000 BC in western Sudan (Africa) [14]. Plants such as *H. sabdariffa* L. synthesize secondary metabolites known as phytochemical compounds which serve as a defense mechanism against pathogens, among other roles [15,16,17]. Some of these compounds have been isolated and purified, then used in human phytotherapy as a treatment for various diseases [18,19] or as adjuvants in chemotherapy [20,21,22]. *Hibiscus* polyphenol extracts have been the subject of many human clinical studies involving patients with hypertension [23], hyperlipidemia, diabetes or obesity [24], and bacterial multidrug resistance [25]. The main constituents of the extracts are organic acids, anthocyanins, polysaccharides, and flavonoids [26], exerting potent antioxidant and radical scavenging activities [24], provided mainly by the presence of high anthocyanin concentrations [13,27]. The anthocyanins belong to the flavonoid class of molecules and are synthesized via the phenylpropanoid pathway. They are synthesized in all tissues of higher plants, including leaves, stems, roots, flowers, and fruits [24].

Furthermore, it has been demonstrated that plant extracts in general and *H. sabdariffa* L. anthocyanin extracts in particular improve and support gut microbial development and protect newly ingested LAB from harsh gastrointestinal conditions [26,28]. Gut microbiota and polyphenols interact in a two-way manner. On the one hand, the polyphenols seem to modulate the gut microbiota variety and metabolic functionality and on the other hand, the gut microbiota degrades polyphenols into active metabolites [29]. At the same time, the enzymatic equipment of gut microbiota improves antioxidant activity by bio-transforming vegetal extracts, thus increasing their bioavailability and functionality [12,28]. By using microbe-mediated bio-transformation of plant extracts, we can enhance their potential to promote health benefits.

To the best of our knowledge, there are no published data on the antioxidant properties of LAB probiotic media or the enhancement of their antioxidant activity by supplementing the culture medium with anthocyanins extracted from *Hibiscus sabdariffa* L. Therefore, in the present study we investigated for the first time the antioxidant activity of the entire probiotic culture medium. The *Weissella confusa* PP29 strain was incubated for four days in a Man–Rogosa–Sharpe (MRS) culture medium containing anthocyanins from *H. sabdariffa* L. in different concentrations. The antioxidant activity of the fermented culture medium was determined by using eight different analysis methods: total phenolic content, ABTS and DPPH radical scavenging assays, hydroxyl radical and superoxide anion radical scavenging ability, ferrous ions’ chelating activity, FRAP assay, and lipid peroxidation inhibitory assay.

## 2. Materials and Methods

### 2.1. Hibiscus sabdariffa *L*. Extract and Microorganism Stock Culture

The *Hibiscus sabdariffa* L. flowers used were from a native Romanian culture plant, representing a commercial tea for internal use, and were purchased from a store of natural products. The anthocyanins from the *Hibiscus sabdariffa* L. flowers were extracted using the same starting material and identical procedure as previously published by our group (Anghel et al. in 2021 [1] and Dimofte et al. in 2022 [26]). Briefly, we obtained an acidified hydroalcoholic extract of dried flowers which was filtered in order to purify anthocyanins, then the extract was dried under vacuum. The extract was analyzed using high-performance liquid chromatography (HPLC) technique for the anthocyanin quantification with cyanidin-3-sambubioside and delphinidin-3-sambubioside as standards for identifying the components of the *Hibiscus sabdariffa* L. flower extract, and the results were published previously by our group [1,26].

The LAB strain coded PP29, previously identified as *Weissella confusa* species by 16S rRNA gene sequence analysis [30], was used for fermentations from a stock stored at −80 °C in MRS with 20% glycerol (both purchased from Merck Group, Darmstadt, Germany), after activation.

### 2.2. Fermentation Conditions

The basic culture medium was denoted as M0 and contained MRS (55.3 g/L) supplemented with peptone (5 g/L) and sucrose (80 g/L). The experimental culture media were supplemented with two concentrations of *H. sabdariffa* aqueous extract: 1000 or 2000 μg anthocyanins/mL media (denoted as M1 and M2, respectively). The codifications and descriptions of the studied samples are presented in Table 1. The fermentation conditions were previously explained in detail in [26].

### 2.3. In Vitro Antioxidant Activity Evaluation of the Fermented Culture Media

All the samples were heat-inactivated after fermentation [26], the biomass was harvested by centrifugation at 4900× *g* for 15 min, and the supernatant was kept at 4 °C until use. The in vitro antioxidant activity evaluation was performed without prior dilution on fresh culture media (T0) and on culture media stored at 4 °C in sterile conditions for 6 months (T6). The antioxidant activities of all the fermented samples were compared with those obtained for L-ascorbic acid (AA), as the antioxidant reference, and the two anthocyanin extracts prepared in water (A1 and A2). All experiments were performed in triplicate. Each test was finished by reading the respective absorbance in 100 μL reaction mix placed in a 96-well plate in triplicate, using a FLUOstar^®^ Omega Microplate Reader (BMG LABTECH, Ortenberg, Germany). All reagents used were purchased from Sigma-Aldrich Chemie GmbH, Taufkirchen, Germany.

The sample antioxidant activity (SAA) preservation after 6 months of storage (T6) for each employed method was calculated using the following formula:

$$SAA\;preservation\left(\%\right)=\frac{SAA\;at\;T6}{SAA\;at\;T0}\times 100$$

#### 2.3.1. Total Phenolic Content (TPC)

The TPC was evaluated by Folin–Ciocalteu’s (FC) assay as follows: 150 μL of each sample (distilled H_2_O used as a blank) was mixed with 750 μL of FC mix (1:9, *v*/*v*; FC/distilled H_2_O) and 600 μL of Na_2_CO_3_ (75 g/L). The reaction was left for 2 h in the dark and absorbance was measured at 760 nm. The TPC is expressed as gallic acid equivalents (μg GAE/mL sample) using a calibration curve in the 0–80 μg gallic acid/mL range [31]. 

#### 2.3.2. ABTS Radical Scavenging Assay

The samples’ ABTS^+^• scavenging properties were performed as previously described by Petrovici et al. [31]. In short, 1.2 mL of ABTS^+^• reagent was mixed with 0.3 mL of each sample, AA as a reference, or 95% ethanol as a negative control, incubated for 6 min, then the absorbance was measured at 734 nm. The ABTS^+^• reagent was obtained by mixing 0.35 mL of 7.4 mM ABTS diammonium salt with 0.35 mL of 2.6 mM potassium persulfate, kept in the dark at room temperature for 16 h, then diluted 1:50 with 95% ethanol. The samples’ ABTS^+^• inhibition percentages were calculated using the following formula:

$${ABTS}^{+}•Inhibition\;\%=\left(1-\frac{As}{A0}\right)\times 100$$

where As is the sample absorbance and A0 is the blank absorbance [31].

#### 2.3.3. DPPH Radical Scavenging Assay

Following the procedure published by Petrovici et al. [31], 1000 μL DPPH (89.7 μM in methanol) was mixed very well with 500 μL of each sample, AA as a reference, or methanol as a negative control, then incubated in the dark for 2 h. After the reaction was complete, the absorbance was measured at 517 nm. The DPPH• inhibition percentages of the samples were calculated by using the following formula:

$$DPPH•Inhibition\;\%=\left(1-\frac{As}{A0}\right)\times 100$$

where As is the sample absorbance and A0 is the blank absorbance [31].

#### 2.3.4. Hydroxyl Radical (HO•) Scavenging Ability

The HO• scavenging ability was determined as follows: 400 μL of sample, AA as a reference, or distilled H_2_O as a control, was mixed vigorously with 400 μL O-phenanthroline (2.5 mM) and 400 μL PBS, 0.2 M, pH 7.4. Then, 400 μL of ferrous sulfate heptahydrate (2.5 mM) and 400 μL of H_2_O_2_ were added and the mixture was incubated at 37 °C on a thermoshaker for 1 h. After incubation, the absorbance was measured at 536 nm. The HO• scavenging percentage was calculated with the following formula:

$$Scavenging\;rate\left(\%\right)=\left(\frac{As-A1}{A0-A1}\right)\times 100$$

where As is the absorbance of the sample; A0 is the absorbance of distilled water in the reaction; and A1 is the absorbance of hydrogen peroxide in the reaction [32].

#### 2.3.5. Superoxide Anion Radical (O_2_^−^•) Scavenging Activity

The superoxide anion radical (O_2_^−^•) scavenging activity of the studied samples was determined as follows: 40 μL of sample, AA as a reference, or distilled H_2_O as a control, was mixed with 1.8 mL Tris–HCl (0.05 M, pH 8) and incubated for 20 min at 25 °C. Next, 160 μL of pyrogallol (25 mM) was added and the mixture was incubated at 25 °C for 5 min. Then, 10 μL HCl (8 M) was added to stop the reaction and the absorbance was measured at 325 nm. The scavenging percentage was calculated using the following formula:

$$Scavenging\;rate\left(\%\right)=\left(1-\frac{As}{Ac}\right)\times 100$$

where Ac is the control’s absorbance and As is the sample’s absorbance [33].

#### 2.3.6. Ferrous Ions’ (Fe^2+^) Chelating Activity

The chelating activity of the studied samples was compared to AA (as a common antioxidant reference) and was estimated using a previously described method [32]. In brief, 200 μL of sample, AA, or distilled H_2_O as a control, and 100 μL ferrous chloride tetrahydrate (2 mM) were mixed. Then, 200 μL ferrozine (2 mM) was added and the total volume was adjusted to 2 mL with ethanol. The mixture was vigorously shaken and incubated at room temperature for 10 min. The Fe^2+^ chelating activity was estimated by measuring the absorbance of the Fe^2+^–ferrozine complex at 562 nm. The inhibition percentage of the Fe^2+^–ferrozine complex was calculated using the following formula:

$${Fe}^{2+}chelating\;effect\left(\%\right)=\left(1-\frac{As}{Ac}\right)\times 100$$

where Ac is the control’s absorbance and As is the sample absorbance [32].

#### 2.3.7. Ferric Ions’ (Fe^3+^) Reducing Antioxidant Power (FRAP) Assay

A FRAP assay was performed for all the studied samples, AA, and distilled H_2_O, using a protocol adapted and published previously [32]. A total of 50 μL of sample was mixed with 650 μL sodium phosphate buffer (Na_2_HPO_4_/KH_2_PO_4_, 0.2 M, pH 6.6) and 650 μL potassium ferricyanide 1% and the mixture was incubated for 20 min at 50 °C. After 20 min, 650 μL trichloroacetic acid (10%) was added to the mixture and 910 μL of this solution was mixed with 910 μL distilled H_2_O and 180 μL ferric chloride (0.1%). The absorbance was measured at 700 nm. The reducing power (%) was expressed as the ratio between the absorbance of the sample and the highest recorded absorbance.

#### 2.3.8. Lipid Peroxidation Inhibitory Assay

Lipid peroxidation inhibitory activity was measured using a previously described method [32] as follows: 100 μL of sample, AA as a reference, or distilled H_2_O as a control was mixed with 900 μL phosphate buffer (dipotassium hydrogen phosphate 0.2 M at pH 7) and 1000 μL linoleic acid emulsion (prepared by mixing 155 μL linoleic acid and 175 μg Tween 20 in 50 mL phosphate buffer, 0.2 M). The mixture was incubated at 37 °C for 24 h, then 50 μL of this solution were mixed with 1.85 mL ethanol and 50 μL FeCl_2_ solutions (20 mM in 3.5% HCl). The resulting solution was mixed thoroughly and 50 μL of potassium thiocyanate (30%) was added. The absorbance of the resulting clear solution was recorded at 500 nm. The inhibitory effect was calculated using the following formula:

$$Inhibitory\;effect\left(\%\right)=\left(1-\frac{As}{Ac}\right)\times 100$$

where As is the absorbance of the sample and Ac is the control’s absorbance [32].

### 2.4. Statistical Analysis

GraphPad Prism 8 software (GraphPad Software Inc., San Diego, CA, USA) was used for statistical analysis. Data are represented graphically as means ± standard error of the mean. One-way ANOVA with Dunnett’s test for multiple comparisons was used, comparing all samples at T0 to AA and considering *p* < 0.05 as statistically significant.

## 3. Results and Discussion

Recently, we published a study investigating the effect of *Hibiscus sabdariffa* L. anthocyanin extract on the probiotic and prebiotic properties of *Weissella confusa* in order to recommend the fermented media as a prebiotic and probiotic supplement [26]. We found that the fermented media presented high prebiotic and probiotic properties correlated to high anthocyanin concentration, as well as conferred protection of LAB from low pH and bile salt [26]. Due to these findings and the fact that natural antioxidants have high potential to scavenge free radicals [34], in the present study, we investigated the antioxidant activity of the fermented culture media.

To the best of our knowledge, there have been no studies conducted on the antioxidant activity of LAB fermented media. In the present study, we performed fermentations of the *W. confusa* PP29 strain in culture media with different anthocyanin concentrations from *Hibiscus sabdariffa* L. extract. The antioxidant activity of the whole culture medium was determined through eight analysis methods optimized in our laboratory. 

After the fermentation process was completed, the samples were heated to 100 °C for 20 min, in order to stop culture growth and inactivate the characteristic enzymatic activity. The fermentation medium was separated from the biomass by centrifugation, and the antioxidant activity was determined for all samples of fresh culture media (T0). After 6 months storage at 4 °C (T6), the culture media were tested again in order to determine the preservation of antioxidant activity during storage. The antioxidant activities of all the fermented samples at T0 were compared with those obtained for AA and the two anthocyanin extracts prepared in water.

### 3.1. Total Phenolic Content (TPC)

**Figure 1:**
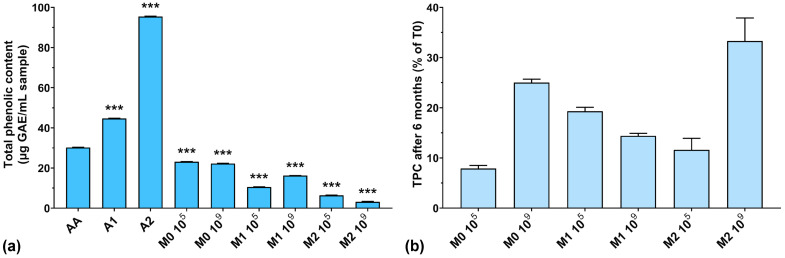
(**a**) Total phenolic content of the studied samples expressed as gallic acid equivalents (GAEs) at T0; (**b**) Total phenolic content preservation after 6 months of storage (T6). *** *p* < 0.001 vs. AA, One-way ANOVA with Dunnett’s test for multiple comparisons.

In the first phase, the TPC of all samples was tested considering the addition of anthocyanin extract in the culture medium. As can be seen from Figure 1a, the highest amount of TPC was recorded for unfermented extracts (44.7 μg/mL GAE for A1, and 95.4 μg/mL GAE for A2, respectively). The TPC in the fermented samples was very low after 4 days of lactic fermentation. At the same time, the TPC of fermented culture media with higher anthocyanin concentrations was much lower than those with low concentrations of anthocyanins. Also, if we look at our previously published results regarding biomass yield obtained in the same culture media, we can see that a higher anthocyanin content positively influences the growth of LAB (11.2 g/L dry biomass for 2000 μg anthocyanins/mL vs. 7.8 g/L dry biomass for 1000 μg anthocyanins/mL) [26]. These results suggest that LAB use anthocyanins as a carbon source. This aspect was also demonstrated in previously published works in which yeasts used polyphenolic compounds as a growth source [35,36]. On the other hand, Endo et al. demonstrated that in aerated cultures with characteristic LAB culture media, *Weissella cibaria* produces large concentrations of hydrogen peroxide [37], which has been shown to degrade anthocyanins from culture media [38], thus reducing the TPC. However, these observations require further in-depth studies on the structural degradation of anthocyanins.

Also, Huang et al. reported that by using microbial fermentation, they were able to increase the concentration of natural antioxidants in the media [28]. This could be explained by the enzymatic cleaving of polyphenolic glycosides by microorganisms, which then use sugar residues as a carbon source, while polyphenols are released in the culture media, thus increasing the TPC. The resulting aglycones have higher antioxidant activity and nutraceutical activity compared to their corresponding glycosides [28]. Although anthocyanins are very sensitive to degradation by several factors such as pH, temperature, light, and oxygen [39], we observed that their properties seemed to be preserved in the fermentative culture medium.

Figure 1b shows the percentages of TPC preservation after 6 months of storage. It can be observed that the TPC was lower at T6 compared to T0 (below 35% preservation), which leads us to think that the structure of the anthocyanins was degraded over time, either through spontaneous chemical processes (such as oxidation or reduction) or by the remaining enzymatic systems in the culture medium not inactivated by the heating treatment. Following this degradation process, the groups that normally react with the FC reagent were inactivated or detached, with the remaining structure thus rendered inactive from this point of view. A low TPC preservation does not necessarily mean a lower antioxidant activity of the culture medium, but only that the remaining structures are no longer reactive with the FC reagent. The best reactivity after 6 months of storage can be observed for the M2 10^9^ experiment, which had the highest initial anthocyanins concentration and the highest inoculum.

### 3.2. ABTS Radical Scavenging Activity

**Figure 2:**
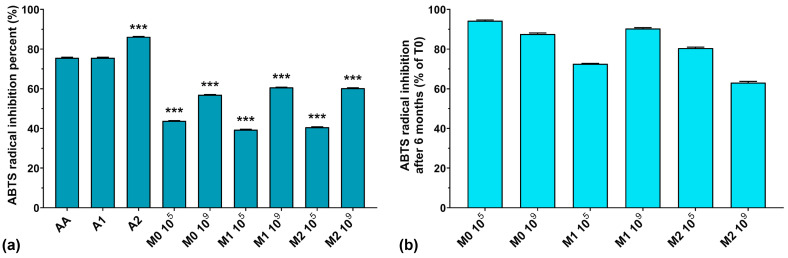
(**a**) ABTS radical inhibition percentages of the studied samples at T0; (**b**) ABTS radical inhibition preservation after 6 months of storage (T6). *** *p* < 0.001 vs. AA, One-way ANOVA with Dunnett’s test for multiple comparisons.

The ABTS assay results are graphically represented in Figure 2a. As can be seen, the highest ABTS radical scavenging activity was recorded for A1 > A2 > AA, with values of 86.2, 75.6, and 75.6%, respectively. The fermented media without anthocyanins inoculated with 10^5^ CFU/mL resulted in slightly higher ABTS radical scavenging activity values compared to the corresponding media containing anthocyanins. At the same time, with the increase in the initial inoculum in the fermentation processes, the ABTS radical scavenging activity also increased, regardless of anthocyanins’ presence in the culture medium. Recently, Pei et al. and Li et al. both demonstrated that EPS isolated from culture media have strong ABTS radical scavenging activity [40,41]. However, we previously demonstrated that *W. confusa* PP29 strain fermentation inoculated with 10^5^ CFU/mL produced more EPS compared to its 10^9^ CFU/mL counterpart [26]. Observing these facts, we can conclude that the ABTS radical scavenging activity of the fermented media increased along with the number of CFU/mL in the initial inoculum, but was independent of the biosynthesized EPS amount present in the media.

It is interesting to note that the ABTS radical inhibition activity was reduced by less than 20% after 6 months of storage at 4 °C (as depicted in Figure 2b), except for M1 10^5^ and M2 10^9^ fermentations, which exhibited losses of activity of 27.4% and 36.9%, respectively. This suggests that finding equilibrium between the initial inoculum and the anthocyanin concentration in the culture medium is crucial for maintaining long-term preservation of the antioxidant properties of LAB fermented media. 

### 3.3. DPPH Radical Scavenging Assay

**Figure 3:**
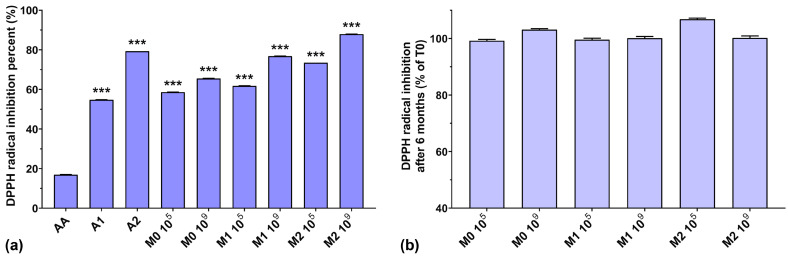
(**a**) DPPH radical inhibition percentages of the studied samples at T0; (**b**) DPPH radical inhibition preservation after 6 months of storage (T6). *** *p* < 0.001 vs. AA, One-way ANOVA with Dunnett’s test for multiple comparisons.

As can be observed in Figure 3a, the DPPH radical scavenging activities of the anthocyanin extracts (A1 and A2) were higher than that of AA. Similar to the ABTS radical scavenging activity, the DPPH radical scavenging activity of the fermented media increased slightly when a higher initial inoculum was used (by 7–15%). Taking into consideration that EPS isolated from culture media have very good DPPH radical scavenging activity [40], dependent on their concentration [41], and that the biosynthesized EPS yield is inversely proportional to the initial inoculum [26], this suggests that other compounds other than EPS, present in the fermented medium, have a distinct impact on DPPH radical scavenging activity. Furthermore, we can also note a gradual increase in the scavenging activity of the fermented media (by up to 22.4%), dependent on the anthocyanin concentrations used. It is interesting to note that the DPPH radical scavenging activity of AA was very low (16.8%) compared to all fermented samples, even those without anthocyanin supplementation, although AA is popularly acclaimed as a potent antioxidant and free radical scavenger [42]. From Figure 3b, we can observe that storage over a 6-month period did not significantly affect the DPPH radical scavenging activity of the fermented media.

### 3.4. Hydroxyl Radical (HO•^−^) Scavenging Ability

**Figure 4:**
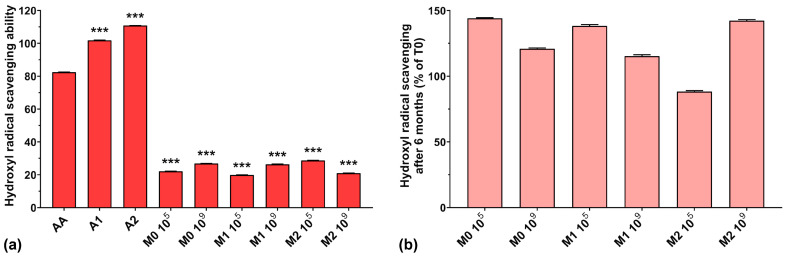
(**a**) Hydroxyl radical (HO•^−^) scavenging ability of the studied samples at T0; (**b**) Hydroxyl radical scavenging preservation after 6 months of storage (T6). *** *p* < 0.001 vs. AA, One-way ANOVA with Dunnett’s test for multiple comparisons.

The hydroxyl radical scavenging activities of AA, A1, and A2 were very high, with values of 82.4%, 101.8%, and 110.8%, respectively (Figure 4a). The results obtained for all fermented samples were below 30%, regardless of anthocyanins’ presence in the culture medium. A possible explanation could be that microorganism fermentation can eliminate excessive hydroxyl radicals produced in the early growth stage in the culture medium, ensuring a normal cellular growth [43], suggesting a depleted capacity of fermented media to scavenge hydroxyl radicals. However, somewhat in contrast to our findings, it has been demonstrated that EPSs are able to scavenge hydroxyl radicals, in a concentration-dependent manner, with higher efficiency compared to AA [7,40,41]. 

Figure 4b shows the preservation percentages of the hydroxyl radical scavenging activity of the fermented media after 6 months of storage. We can observe an increase in scavenging activity of approximately 40% for the M0 10^5^, M1 10^5^, and M2 10^9^ samples and an increase of over 15% for the M0 10^9^ and M1 10^9^ samples. Only the M2 10^5^ sample experienced a 12% loss of hydroxyl radical scavenging capacity over 6 months of storage. As in the case of ABTS radical scavenging activity, this suggests the need for properly balancing the initial inoculum and the anthocyanin concentration in the culture medium in order to maintain the long-term preservation of the antioxidant properties of LAB fermented media.

### 3.5. Superoxide Anion Radical (O_2_^−•^) Scavenging Activity

The superoxide anion radical concentration in bacterial cells normally increases during the fermentative process, but enzymes present in the culture media are capable of neutralizing them. The scavenging of this radical is crucial for normal LAB growth because O_2_^−•^ inactivates glutathione peroxidase and catalase in the culture media [43], leading to undesired enzyme imbalances which affect all bacterial metabolic processes.

**Figure 5:**
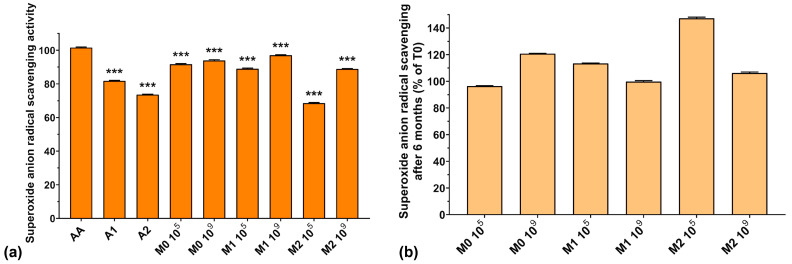
(**a**) Superoxide anion radical scavenging activity of the studied samples at T0; (**b**) Superoxide anion radical scavenging preservation after 6 months of storage (T6). *** *p* < 0.001 vs. AA, One-way ANOVA with Dunnett’s test for multiple comparisons.

Figure 5a presents the superoxide anion radical scavenging activities of the analyzed samples. We can observe that the values obtained for the fermented media are comparable to those of AA. However, the superoxide anion radical scavenging activity of A2 and of the fermented media supplemented with A2 (73.6% for A2, 68.5% for M2 10^5^, and 88.9% for M2 10^9^) was lower compared to the fermented media without anthocyanin extract (91.7% for M0 10^5^ and 93.9% for M0 10^9^) or to samples with a lower anthocyanin concentration—1000 μg/mL (81.7% for A1, 89.0% for M1 10^5^, and 89.1% for M1 10^9^). At the same time, the fermented medium without anthocyanin supplementation exhibited higher superoxide anion radical scavenging activity compared to the anthocyanin extracts (93.9% for M0 10^9^ compared with 81.7% for A1 and 73.6% for A2). This is in agreement with Pei et al. who demonstrated recently that EPSs isolated from culture media have high O_2_^−•^ radical scavenging activity [40]. Furthermore, the fermented samples containing either anthocyanin extract showed higher superoxide anion radical scavenging activity compared to the anthocyanin extract by itself (see Figure 5a). From these results, we can conclude that the fermented media are more effective in neutralizing superoxide anion radical than the anthocyanin extracts, by virtue of their components, suggesting that LAB fermentation enhances the antioxidant activity of the final product.

Figure 5b shows the preservation of the scavenging capacity of the samples over 6 months of storage. As we observed in the case of HO^–^ radical activity, the superoxide anion radical scavenging activity improved after 6 months of storage by 6–47%, which is encouraging for potential food and biomedical applications. 

### 3.6. Ferrous Ions’ (Fe^2+^) Chelating Activity

Ferrous ions have an important role in hydroxyl radical generation in the Fenton reaction and it has been shown that ferrous ions’ chelating ability can be enhanced by microbial fermentation [43].

**Figure 6:**
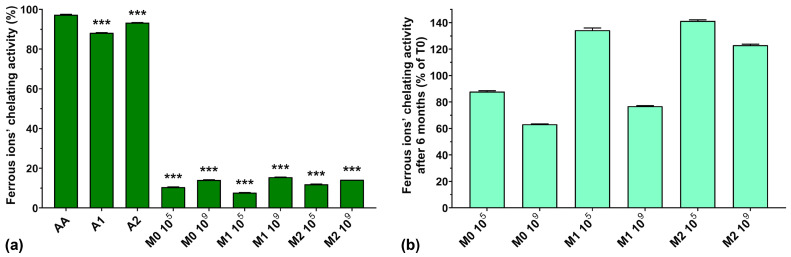
(**a**) Ferrous ions’ chelating activity of the studied samples at T0; (**b**) Ferrous ions’ chelating activity preservation after 6 months of storage (T6). *** *p* < 0.001 vs. AA, One-way ANOVA with Dunnett’s test for multiple comparisons.

From Figure 6a, we can see that the ferrous ions’ chelating activity was higher for AA compared with the two anthocyanin extracts (97.2% for AA compared with 88.2% for A1 and 93.3% for A2). Normally, EPSs biosynthesized by LAB exhibit metal chelating activity [10], but the fermented samples analyzed in this study showed very low ferrous ion chelating activity (less than 16%), independent of anthocyanin supplementation and concentration. This could suggest a depleted capacity of the fermented media to chelate ferrous ions due to the fermentation process itself, as seen in the case of hydroxyl radical scavenging activity described above.

Figure 6b shows a graphic representation of the ferrous ions’ chelating activity preservation after 6 months of storage. We can observe that the chelating activity increased after 6 months of storage for the M1 10^5^, M2 10^5^, and M2 10^9^ samples by 34.3, 41.3, and 23%, respectively. However, storage diminished the chelating activity of the M0 10^5^, M0 10^9^, and M1 10^9^ samples to 88.0, 63.2, and 76.8%, respectively, of their initial values.

### 3.7. Ferric Ions’ (Fe^3+^) Reducing Antioxidant Power (FRAP) Assay

**Figure 7:**
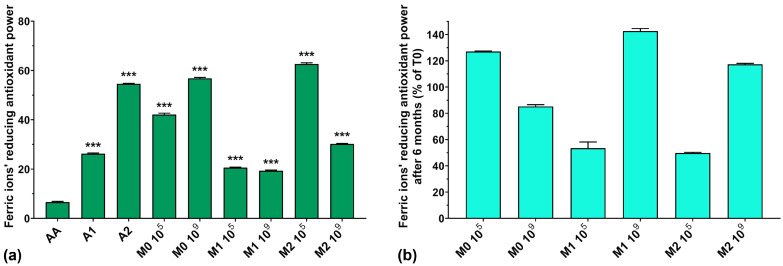
(**a**) Ferric ions’ reducing antioxidant power (FRAP) of the studied samples at T0; (**b**) FRAP preservation after 6 months of storage (T6). *** *p* < 0.001 vs. AA, One-way ANOVA with Dunnett’s test for multiple comparisons.

The FRAP assay results for all analyzed samples are graphically represented in Figure 7a. The FRAP of AA is very low compared to the anthocyanin extracts and the fermented media samples. The simple culture medium inoculated with 10^9^ showed higher FRAP activity compared to samples inoculated with 10^5^ (56.7% for M0 10^9^ compared with 42.1% for M0 10^5^). Overall, the FRAP of the fermented samples does not follow an expected correlation with the increase in the concentration of anthocyanins in the culture medium. The FRAP of the extracts increased with anthocyanin concentration (26.1% for A1 and 54.6% for A2), but in the culture medium, their activity was much lower (19.3% for M1 10^9^ compared with 30.2% for M2 10^9^). An exception to this statement was noted for M2 10^5^, where the FRAP was higher than that of the extract used in the preparation of the culture medium (62.6% for M2 10^5^ compared with 54.6% for A2). Regarding the preservation of the FRAP over 6 months of storage, it can be seen from Figure 7b that the samples containing anthocyanin extract and inoculated with 10^9^ showed increased FRAP with storage (143% for M1 10^9^ and 117% for M2 10^9^) compared to samples inoculated with 10^5^, which showed poor preservation of FRAP (53% for M1 10^5^ and 50% for M2 10^5^).

### 3.8. Lipid Peroxidation Inhibitory Assay

In lipid peroxidation processes, alkoxyl radicals and organic hydroperoxides are generated and generally bound to proteins in living cells [4]. Vo et al. recently showed that anthocyanins inhibit lipid peroxidation and are also capable of superoxide anion radical and hydroxyl radical scavenging activity [44]. Due to their deprotonated structure in aqueous solutions, the radical scavenging activity of anthocyanins occurs as a regeneration cycle, increasing their protective effects against oxidative stress [23,44].

**Figure 8:**
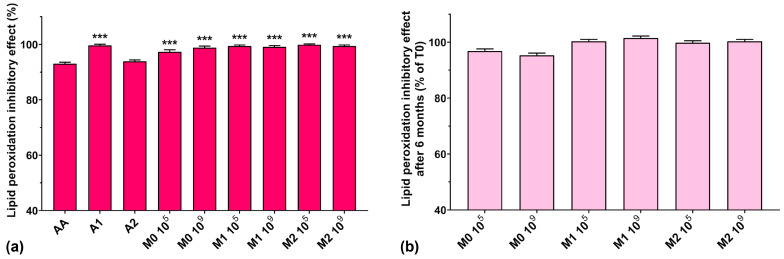
(**a**) Lipid peroxidation inhibitory activities of the studied samples at T0, determined at 24 reaction hours; (**b**) Lipid peroxidation inhibitory effect preservation after 6 months of storage (T6). *** *p* < 0.001 vs. AA, One-way ANOVA with Dunnett’s test for multiple comparisons.

The lipid peroxidation inhibitory activity for all studied samples is represented in Figure 8a. We can observe that all analyzed samples had similar activity compared to AA, which, incidentally, exhibited the lowest value (93%), although it is known to prohibit lipid peroxidation and protect cells from oxidative damage caused by ROS [43]. Fermented media not supplemented with the anthocyanin extract showed a higher lipid peroxidation inhibitory activity compared to the most concentrated anthocyanin extract (97.3% for M0 10^5^ and 98.8% for M0 10^6^ compared with 93.9% for A2). All fermented media samples supplemented with anthocyanins showed a lipid peroxidation inhibitory activity of over 99%. Furthermore, we observed that all samples maintained their lipid peroxidation inhibitory activity at approximately 100% after 6 months of storage (Figure 8b).

## 4. Conclusions

The inherent antioxidant potential of anthocyanins, combined with the probiotic properties of *Weissella confusa* PP29, create a unique synergy that warrants exploration. Furthermore, the bioactive compounds derived from *Hibiscus sabdariffa* L. add a layer of complexity, potentially bestowing additional health benefits beyond antioxidation.

In the present study, we investigated for the first time the antioxidant activity of the entire probiotic culture media of *Weissella confusa* PP29 and the effect of *H. sabdariffa* L. anthocyanin extracts on their antioxidant properties. The DPPH radical scavenging and FRAP of the fermented products was higher compared to ascorbic acid and antioxidant extracts, while their superoxide anion radical scavenging and lipid peroxidation inhibitory activity were comparable to that of ascorbic acid. The overall antioxidant properties of the fermented media were enhanced by the addition of anthocyanins before fermentation and were preserved over 6 months of storage. 

The augmented antioxidant efficacy of the enriched culture medium holds immense promise for both functional food development and potential health applications. This innovative approach opens avenues for the creation of antioxidant-rich probiotic products that can contribute to maintaining overall well-being and reducing the risk of chronic diseases. Further research and exploration are warranted to fully elucidate the scope of this innovative approach and its broader implications for human health.

## Figures and Tables

**Table 1:** The codifications and descriptions of the studied samples.

Sample Code	μg Anthocyanins/mL Culture Media	Description
A1	1000	Anthocyanins extracted from *H. sabdariffa*—1000 μg/mL in water
A2	2000	Anthocyanins extracted from *H. sabdariffa*—2000 μg/mL in water
M0 10^5^	–	PP29 strain fermented in M0 inoculated with 10^5^ CFU/mL
M0 10^9^	–	PP29 strain fermented in M0 inoculated with 10^9^ CFU/mL
M1 10^5^	1000	PP29 strain fermented in M1 inoculated with 10^5^ CFU/mL
M1 10^9^	1000	PP29 strain fermented in M1 inoculated with 10^9^ CFU/mL
M2 10^5^	2000	PP29 strain fermented in M2 inoculated with 10^5^ CFU/mL
M2 10^9^	2000	PP29 strain fermented in M2 inoculated with 10^9^ CFU/mL

## Data Availability

The data presented in this study are available upon request from the corresponding author.
